# Washing Amalgams

**Published:** 1880-08

**Authors:** Thos. Fletcher


### ARTICLE VI.

### *Washing Amalgams.*

BY THOS. FLETCHER.

I have so frequently been told by operators of their great success with washed amalgams as compared with unwashed, that I have been at some pains and trouble to discover the reason of what may be granted as a fact. Knowing with certainty that the process of washing was a serious evil, the fact of practice being directly contrary to theory, puzzled me not a little until I watched a good operator at work washing an amalgam, when the explanation of the anomaly was simple enough. It would appear that all who have compared washed and unwashed amalgams have treated both in the same way, *i. e.*, by mixing with excess of mercury and squeezing out. Knowing that without exception all plastic fillings are liable to total failure unless they set rapidly and thus preventing alteration of form by moisture, and knowing also that amalgams mixed with excess of mercury are exceedingly slow in setting, the advantage of washing was explained in the simplest manner by the longer time required to manipulate the mass, which resulted in the filling being harder than when finished than if no time had been lost by the washing process.

As the mixing with excess of mercury had been utterly condemned both by theory and practice, this apparent advantage in favor of washing is at last proved to be a false one.

Any amalgam to be absolutely reliable must be worked in such a manner that it is hard enough for mastication as soon as finished, or at least in a few minutes after. Granting this as a necessity for good work, the process by which it is obtained and which requires the smallest quantity of mercury is the best.—*Dental Advertiser.*

### *Virginia State Dental Association.*

The regular annual meeting of the Virginia State Dental Association will be held in the city of Richmond, Tuesday, December 14th, 1880, at 10 o'clock A. M.

It is hoped that every member of the Association will use his best endeavors to be present, and lend his aid in making the meeting interesting and profitable.

All members of the profession, not only in Virginia, but in sister States, whether members of the Society or not, are cordially invited to meet with us and take part in our discussions.

As there was not a quorum present at the last meeting of the Association, the President has decided to retain the same committees. The members of the committees are respectfully requested to give strict attention to the extract from the Constitution.

Members and visitors to the Association will call at the office of the Corresponding Secretary upon arrival for information as to place of meeting.

Fraternally yours,
L. M. Cowardin,
*Corresponding Secretary.*

---

### *American Academy of Dental Science.*

The thirteenth annual meeting of the American Academy of Dental Science, will be held in Boston, on Wednesday, October 27th, at 10 o'clock A. M.

The annual address will be delivered at 2 o'clock P. M. by Dr. Joshua Tucker of Boston. Dr. Tucker's world-wide reputation and large experience cannot fail to make the occasion both interesting and instructive. Members of the profession are cordially invited to attend.

C. P. Wilson,
*Corresponding Secretary.*

